# Measuring Childlessness Among Middle-Aged and Older Americans

**DOI:** 10.1093/geroni/igaa057.081

**Published:** 2020-12-16

**Authors:** Xiao Xu, Jersey Liang, BoRin Kim, James Raymo, Mary Beth Ofstedal

**Affiliations:** 1 Yale University, New Haven, Connecticut, United States; 2 University of Michigan, Ann Arbor, Michigan, United States; 3 University of New Hampshire, Durham, New Hampshire, United States; 4 Princeton University, Princeton, New Jersey, United States

## Abstract

Existing literature on childlessness among middle-aged and older Americans is sparse, and measuring childlessness is not straightforward for those with complex family histories. To address this knowledge gap, we examined data on 19,929 respondents age ≥50 from the 2016 Health and Retirement Study. All analyses accounted for complex sample design to generate nationally representative estimates. The proportion of respondents without children differed significantly depending on how “childless” was defined: 1) 14.9% (95% confidence interval [CI]: 13.9-15.9%) having no biological children, versus 2) 10.4% (95% CI: 9.5-11.3%) having no children/step-children that were living and in-contact. When measured based on absence of biological children, the prevalence of childlessness was higher in younger cohorts (17.7%, 13.2%, and 9.0% for age 50-64, 65-74, and ≥75 years, respectively, p<0.001) and among more educated individuals (17.4%, 12.3%, and 9.6% for more than high school, high school, and less than high school education, respectively, p<0.001). The prevalence of childlessness was also higher among men (16.7%) than women (13.2%) (p<0.001) and among non-Hispanic whites (16.0%) than Hispanics (9.8%) (p<0.001). Similar patterns, but lower prevalence, were observed when measuring childlessness based on absence of children/step-children that were living and in-contact. Although non-Hispanic whites (16.0%) were more likely than non-Hispanic blacks (13.0%) to have no biological children (p=0.007), a similar proportion of them had no children/step-children that were living and in-contact (10.8% versus 10.6%, p=0.06). Given fertility decline and growing family complexity, these findings help inform the structure of social support and long-term care needs of middle-aged and older Americans.

